# Improving efficacy and maintaining safety in the treatment of alopecia with low-dose oral minoxidil and spironolactone combination therapy: A retrospective review

**DOI:** 10.1016/j.jdin.2024.07.023

**Published:** 2024-08-27

**Authors:** Ambika Nohria, Deesha Desai, Michelle Sikora, Nnaemeka Anyanwu, Avrom Caplan, Jerry Shapiro, Kristen Lo Sicco

**Affiliations:** a Ronald O. Perelman Department of Dermatology, University Grossman School of Medicine, New York, New York; b University of Pittsburgh School of Medicine, Pittsburgh, Pennsylvania; c New York Medical College, Valhalla, New York

**Keywords:** alopecia, androgenetic alopecia, female pattern hair loss, minoxidil, oral minoxidil, spironolactone

*To the Editor:* Low-dose oral minoxidil (LDOM) and spironolactone are popular off-label treatments for female androgenetic alopecia (AGA).^1^ Although both drugs have demonstrated efficacy and safety as monotherapies, clinicians may combine them when results are unsatisfactory.^1^, ^2^, ^3^ However, concerns regarding impacts on blood pressure (BP) may limit their concurrent use. This study aims to evaluate the efficacy and safety of LDOM and spironolactone combination therapy for female AGA.

We performed an institutional review board-approved (i23-00157 approved March 14, 2023) retrospective study of patients seen at NYU Langone Health from January 1, 2008, to August 1, 2023. Inclusion criteria included female sex, AGA diagnosis, use of LDOM or spironolactone, and documented trichometric measurements 12 cm from the glabella. Statistical significance was determined using Wilcoxon rank sum testing with continuity correction or χ^2^ testing.

Our cohort included 21 patients (mean age of 51.3 years and mean LDOM dose of 1.6 mg/day); 12 received LDOM monotherapy (mean age of 58.8 years and mean LDOM of dose 1.9 mg/day), and 9 received a combination therapy with spironolactone (mean age of 41.2 years, mean LDOM dose of 1.3 mg/day, and mean spironolactone dose of 138.9 mg/day). In addition to AGA, 7 patients had concomitant telogen effluvium and 2 had lichen planopilaris. The baseline hair width and density were 60.9 μm and 183.2 hairs/cm^2^ and 64.3 μm and 190.2 hairs/cm^2^ for monotherapy and combination therapy patients, respectively. The average length of time between visits was 134 and 144.0 days for the monotherapy and combination therapy cohorts, respectively. Aside from age (*P* = .03), there were no significant differences in the baseline characteristics between the cohorts. (Table I).

**Table I:** Patient demographics

Charateristic	Overall cohort (n = 21)	Oral minoxidil monotherapy (n = 12)	Oral minoxidil and spironolactone combination therapy (n = 9)	*P*
Age (y)	51.3 ± 18.1	58.5 ± 17.5	41.2 ± 14.1	.03
Alopecia subtype	Androgenetic alopecia: 12 Androgenetic alopecia + telogen effluvium: 7 Androgenetic alopecia + lichen planopilaris: 2	Androgenetic alopecia: 6 Androgenetic alopecia + telogen effluvium: 5 Androgenetic alopecia + lichen planopilaris: 1	Androgenetic alopecia: 6 Androgenetic alopecia + telogen effluvium: 2 Androgenetic alopecia + Lichen planopilaris: 1	
LDOM dose, mg/d	1.7 ± 0.8	1.9 ± 0.9	1.3 ± 0.5	
Spironolactone dose, mg/d	-	-	138.9 ± 48.6	
Length of time between initial and follow-up visit, d	138.3 ± 78.4	134.0 ± 58.5	144.0 ± 103.0	.72
Baseline hair width, μm	63.6 ± 12.4	60.9 ± 12.5	64.3 ± 12.5	.34
Baseline hair density, hairs/cm^2^	186.2 ± 41.6	183.2 ± 49.7	190.2 ± 29.8	.94

All values provided are means with standard deviations.

The average changes in trichometric width and density were 2.3 μm and 1.1 hairs/cm^2^ and 0.8 μm and 13.6 hairs/cm^2^ for the monotherapy and combination therapy cohorts, respectively, with no significant difference in the change for either measurement between cohorts (*P* = .54 and *P* = .36). However, there was a trend toward increased hair density with combination therapy when compared with monotherapy (effect size = 0.21) (Table II).

**Table II:** Efficacy and safety of LDOM monotherapy when compared with LDOM and spironolactone combination therapy

	Overall cohort	Oral minoxidil monotherapy	Oral minoxidil and spironolactone combination therapy	*P*	Effect size
Change in trichometric width	1.2 ± 9.0	2.3 ± 9.9	0.8 ± 8.6	.54	0.14
Change in trichometric density	6.4 ± 24.1	1.1 ± 25.7	13.6 ± 21.1	.36	0.21
Side effects	Total: 8 Dizziness/lightheadedness: 1 Fluid retention/edema: 2 Hypertrichosis: 5	Total: 5 Dizziness/lightheadedness: 0 Fluid retention/edema: 2 Hypertrichosis: 3	Total: 3 Dizziness/lightheadedness: 1 Fluid retention/edema: 0 Hypertrichosis: 2	.66	

The patient that experienced dizziness was a female prescribed LDOM 1.25 mg/day and spironolactone 100 mg/day. No medication changes were required. Changes in trichometric width and density are presented as means with standard deviation.

Eight side effects were reported: 5 from monotherapy and 3 from combination therapy patients. One combination therapy patient experienced lightheadedness or dizziness, which resolved without medication changes (Table II).

This study provides novel insights into LDOM and spironolactone combination therapy. Previous studies on combination therapy have demonstrated positive outcomes, although limited by lower medication doses.^2^^,^^3^ Our work expands on this by investigating outcomes with higher doses of LDOM and spironolactone more commonly employed for AGA treatment. Although not statistically significant, we demonstrated a trend toward improved outcomes with combination therapy with an effect size suggesting that clinical improvement may be present.

Although BP changes were not recorded, only 1 patient experienced dizziness or lightheadedness, which may have resulted from low BP. This tolerability aligns with previous research on the safety of these medications and may provide reassurance to providers who aim to maximize patient outcomes while prioritizing safety.^4^^,^^5^

Limitations of this study include retrospective design, sample size, reliance on self-reported side effects, and lack of BP measurements. Future studies are warranted to fully understand the benefits and risks of combination therapy and identify patients who would most benefit from this approach.

## Conflicts of interest

Dr Sicco has been an investigator for Regen Lab and is an investigator for Pfizer and is a consultant for Pfizer and Aquis. Dr Shapiro is an investigator and consultant for Pfizer and is a consultant for Lilly. Authors Nohria, Desai, Sikora, and Anyanwu and Dr Caplan have no conflicts of interest to declare.
